# The prevalence and antifolate drug resistance profiles of *Plasmodium falciparum* in study participants randomized to discontinue or continue cotrimoxazole prophylaxis

**DOI:** 10.1371/journal.pntd.0007223

**Published:** 2019-03-21

**Authors:** Dennis W. Juma, Peninah Muiruri, Krista Yuhas, Grace John-Stewart, Ronald Ottichilo, John Waitumbi, Benson Singa, Christina Polyak, Edwin Kamau

**Affiliations:** 1 Department of Emerging and Infectious Diseases (DEID), United States Army Medical Research Directorate-Africa (USAMRD-A), Kenya Medical Research Institute (KEMRI) Kisumu, Kenya; 2 Department of Biochemistry, Jomo Kenyatta University of Agriculture and Technology, Nairobi, Kenya; 3 Department of Global Health, University of Washington, Seattle, Washington United States of America; 4 Departments of Global Health, Medicine, Epidemiology, and Pediatrics, University of Washington, Seattle, Washington, United States of America; 5 Centre for Clinical Research, KEMRI, Nairobi, Kenya; 6 U.S. Military HIV Research Program, Walter Reed Army Institute of Research, Silver Spring, MD, United States of America; 7 Henry M. Jackson Foundation for the Advancement of Military Medicine, Bethesda, MD, United States of America; Johns Hopkins Bloomberg School of Public Health, UNITED STATES

## Abstract

**Objective:**

Cotrimoxazole prevents opportunistic infections including falciparum malaria in HIV-infected individuals but there are concerns of cross-resistance to other antifolate drugs such as sulphadoxine-pyrimethamine (SP). In this study, we investigated the prevalence of antifolate-resistance mutations in *Plasmodium falciparum* that are associated with SP resistance in HIV-infected individuals on antiretroviral treatment randomized to discontinue (STOP-CTX), or continue (CTX) cotrimoxazole in Western Kenya.

**Design:**

Samples were obtained from an unblinded, non-inferiority randomized controlled trial where participants were recruited on a rolling basis for the first six months of the study, then followed-up for 12 months with samples collected at enrollment, quarterly, and during sick visits.

**Method:**

*Plasmodium* DNA was extracted from blood specimens. Initial screening to determine the presence of *Plasmodium* spp. was performed by quantitative reverse transcriptase real-time PCR, followed by genotyping for the presence of SP-resistance associated mutations by Sanger sequencing.

**Results:**

The prevalence of mutant haplotypes associated with SP-resistant parasites in *pfdhfr* (51I/59R/108N) and *pfdhps* (437G/540E) genes were significantly higher (P = 0.0006 and P = 0.027, respectively) in STOP-CTX compared to CTX arm. The prevalence of quintuple haplotype (51I/59R/108N/437G/540E) was 51.8% in STOP-CTX vs. 6.3% (P = 0.0007) in CTX arm. There was a steady increase in mutant haplotypes in both genes in STOP-CTX arm overtime through the study period, reaching statistical significance (P < 0.0001).

**Conclusion:**

The frequencies of mutations in *pfdhfr* and *pfdhps* genes were higher in STOP-CTX arm compared to CTX arm, suggesting cotrimoxazole effectively controls and selects against SP-resistant parasites.

**Trial registration:**

ClinicalTrials.gov NCT01425073

## Author summary

Cotrimoxazole, an antifolate, is a fixed-dose trimethoprim-sulfamethoxazole used to prevent opportunistic infections including malaria in HIV-infected individuals. There are concerns that widespread use of cotrimoxazole for prophylaxis may result in selection of *P*. *falciparum* parasites with cross-resistance to other antifolate drugs such as sulphadoxine-pyrimethamine (SP), which is used as intermittent preventive treatment of malaria in pregnancy (IPTp) and in infants (IPTi) in Africa. This sub-study used samples from a clinical trial in which HIV-infected individuals on antiretroviral treatment were randomized to discontinue (STOP-CTX) or continue (CTX) cotrimoxazole prophylaxis for 12 months. The sub-study was designed to assess whether taking cotrimoxazole increased the risk of selecting for parasites with SP-resistant mutations in HIV-infected individuals. Samples were genotyped by sequencing to assess the prevalence of mutations associated with SP-resistance. We found there was no risk of selecting for parasites with SP-resistance mutations while on cotrimoxazole. In fact, the opposite was true; cotrimoxazole controlled parasites carrying SP-resistance mutations as evident by the gradual increase in the prevalence of parasites with mutant alleles in the STOP-CTX arm and not in the CTX-arm. We concluded that cotrimoxazole remains effective in controlling malaria infection despite of the high prevalence of SP-resistant parasites, and its use does not select for SP mutations.

## Introduction

Despite the changes in the epidemiology and improvement in the control of HIV-infection and malaria, both remain important infectious diseases and global health priorities. Through immunosuppression, HIV infection affects the acquisition and persistence of immune response to malaria, causing substantial increase in the malaria prevalence and malaria-related morbidity and mortality [1]. Antiretroviral therapy (ART) and cotrimoxazole, a fixed-dose trimethoprim-sulfamethoxazole (an antifolate) widely used to prevent opportunistic infections in HIV-infected individuals, including falciparum malaria significantly reduces mortality and morbidity in HIV-infected individual. In countries with adequate health infrastructure, the World Health Organization (WHO) recommends daily cotrimoxazole prophylaxis for HIV-infected individuals with low CD4 cell count levels (< 350 cells/mm^3^), whereas in countries with high prevalence of HIV and limited health infrastructure, cotrimoxazole prophylaxis is recommended for all HIV-infected individuals regardless of the CD4 cell count levels [2]. However, there are concerns that widespread use of cotrimoxazole prophylaxis may result in selection of *Plasmodium falciparum* parasites with cross-resistance to closely related antifolate antimalarials such as sulphadoxine-pyrimethamine (SP) [1]. Although artemisinin based combination therapy is the mainstay for treatment of uncomplicated malaria in most malaria endemic countries, SP is widely used as intermittent preventive treatment of malaria in pregnancy (IPTp) and in infants (IPTi) in sub-Saharan Africa (SSA) [3–5].

Some of the important mutant alleles that confer *P*. *falciparum* parasite resistance to SP are in *P*. *falciparum dihydrofolate reductase* (*pfdhfr*) gene at codons 51, 59 and 108, and *P*. *falciparum dihydropteroate synthase* (*pfdhps*) gene at codons 437 and 540. Recent studies have shown high prevalence of these mutant alleles and haplotypes in Western Kenya, including mutant allele at codon 164 in the *pfdhfr* gene which is associated with high-grade resistance to SP [6–9]. Despite the high prevalence of SP-resistant mutations in parasite population in Western Kenya, there is limited clinical evidence associating these mutations with compromised efficacy of cotrimoxazole prophylaxis and IPTp/i [10]. Recent studies have indicated there is fixation of some of the key SP-resistant mutations in the parasite population despite discontinuation of SP as the first-line treatment for more than a decade [6,7,9].

Based on the malaria risk map and the eco-epidemiology of malaria, Kenya is stratified into four malaria ecological regions [11], with the lake region in Western Kenya having the highest, stable transmission of malaria with an estimated prevalence of 27% based on microscopy [12,13] and 37% based on PCR [14]. The HIV-1 prevalence in Kenya is estimated at 5.9%, with Homa Bay County, one of the eight counties in the lake region of Western Kenya having the highest prevalence estimated at 26% [15]. With such high HIV and malaria prevalence, the selective pressure due to cotrimoxazole prophylaxis and the risk of developing antifolate resistance in *P*. *falciparum* warrants further investigation. From February 2012 to August 2013, we conducted a randomized controlled trial (RCT) among adults on ART with evidence of immune recovery to determine whether discontinuation of cotrimoxazole was non-inferior to continuation of cotrimoxazole prophylaxis in decreasing morbidity in Homa Bay County [16]. Study participants were recruited in the first six months of the study on a rolling basis and randomized to discontinue or continue cotrimoxazole, then followed-up for 12 months with the primary endpoint a composite of malaria, pneumonia, diarrhea and non-trauma mortality events. Samples were collected at enrollment, quarterly, and at sick visits which the participants were encouraged to visit the clinic to see study providers for any illness. Malaria was defined as a fever, measured or self-reported, and either a positive rapid diagnostic test or thick smear showing the presence of parasites. Patients who were diagnosed with malaria were treated following the Kenyan Ministry of Health national guidelines. In the RCT study, we found increased incidence of malaria (13.0 in discontinuation of cotrimoxazole arm [STOP-CTX] vs. 0.4 in continuation of cotrimoxazole arm [CTX] per 100 person-years) [16]. In a follow-up study which we characterized the risk associated with stopping CTX therapy by determining parasite density, multiplicity of infecting parasites, and rates of new cases of parasitemia by PCR, malaria incidence was 42.0 in STOP-CTX vs. 9.9 in CTX per 100 person-years [17]. In this study, we determined and compared the prevalence of *P*. *falciparum* parasites with mutations associated with SP-resistance in HIV-infected individuals in the two study population arms, STOP-CTX and CTX.

## Materials and methods

### Ethical considerations

The study protocol was approved by the ethical review committee of the Kenya Medical Research Institute and the institutional review boards of the University of Washington and the Walter Reed Army Institute of Research. All participants gave informed consent. Consent was written if literate and fingerprint if illiterate, with the signature of an independent witness. For the clinical study, Vestergaard Frandsen donated insecticide-treated bednets and water filters. Alere donated cartridges for the Pima machines used for CD4 count measurements.

### Study site and sample collection

Samples used in this study were collected between February 2012 and September 2013 in an unblinded, two-arm randomized non-inferiority clinical trial (clinical trials registration NCT01425073). The details of the study and sample collection have been described elsewhere [16]. Briefly, a total of 500 participants ≥18 years old, HIV seropositive, and taking first-line ART and cotrimoxazole with evidence of immune recovery (ART for ≥18 months and CD4 count > 350 cells/mm^3^) were enrolled in the study, and randomized to discontinue with cotrimoxazole prophylaxis (STOP-CTX; 250 individuals) or continue (CTX; 250 individuals). The study took place in Homa Bay County, Western Kenya, a malaria holoendemic lake endemic region where transmission is intense through-out the year with high annual entomological inoculation rates [12]. Generally, a bimodal pattern of rainfall is observed with the long rainy season from March to June and the short rainy season from November to December, but the periods vary each year with malaria prevalence peaking 1–2 months after the rainy season. Annual rainfall ranges from 700 mm to 1,200 mm with mean temperature of 25°C, with relatively high humidity [14]. This study lasted 18 months, enrollment taking place during the first six months (01 February 2012 to 27 August 2012) with participants randomized to STOP-CTX or CTX. This strategy ensured that participants were enrolled and followed over different malaria seasons.

Blood samples were collected from the participants during the scheduled visits at months 0, 3, 6, 9 and 12 (M0, M3, M6, M9 and M12 respectively), and sick visits. Participants were encouraged to come to the clinic to see study providers for any illness as a sick visit. At each sick visit, a standardized questionnaire was provided to assess participants’ symptoms and a clinician performed a physical exam. Additionally, available and clinically relevant basic diagnostic tests were performed (e.g. malaria smear, chest radiograph, stool ova and parasite exam) to assist with diagnosis as per routine clinic practice. Additionally, pertinent microbiological samples were taken in order to better evaluate cause of illness. If further evaluation was necessary, patients were referred for hospitalization at the nearest facility. Clinical and laboratory records from any hospitalization during participation were reviewed. Participants with malaria were treated following Kenyan national guidelines.

### Genotypic analysis

In the RCT, dried blood spots samples were collected from the participants at enrollment, every 3 months and during sick visits (whether or not they were diagnosed with malaria) for the duration of the study which was 12 months. DNA was extracted from the FTA filter papers using the QIAamp DNA mini kit (Qiagen, Valencia, CA). The detection of *P*. *falciparum* positive samples was performed by quantitative reverse transcriptase real-time PCR (qRT-PCR) as previously described [17,18]. The presence of mutations in *dihydrofolate reductase* (*pfdhfr*: codons 16, 50, 51, 59, 108, and 164) and *dihydropteroate synthase* (*pfdhps*: codons 436, 437, 540, 581, and 613) genes which are associated with antifolate resistance in *P*. *falciparum* samples were assessed by Sanger sequencing as previously described [6]. Briefly, after successfully amplifying the target regions, the PCR amplicons were purified using Exosap-it (Affymetrix, Santa Clara, CA) per the manufacturer’s protocol. Sequencing of the target regions was done on the ABI 3500 xL genetic analyzer using version 3.1 of the big dye terminator method (Applied Biosystems, Foster City, CA). Bioinformatics analysis of the sequence data was done on the CLC Main Work Bench v6 software (Qiagen, Redwood City, California, USA). All sequences were compared against the *pfdhfr* (Accession Number; XM_001351443) or *pfdhps* (Accession Number; XM_001349382) 3D7 reference sequence published at the NCBI database.

### Statistical analysis

The different *Plasmodium* species and genotype polymorphisms within *pfdhfr* and *pfdhps* genes of *P*. *falciparum* were analyzed as proportions showing frequency rates. The differences in frequencies were determined by the Chi-square test. All statistical analyses were performed at the 5% significance level. Graph pad Prism 4.0 software (Graph pad Software, San Diego, California, USA) was used for the analyses.

## Results

### Prevalence of falciparum and non-falciparum malaria

**Fig 1:**
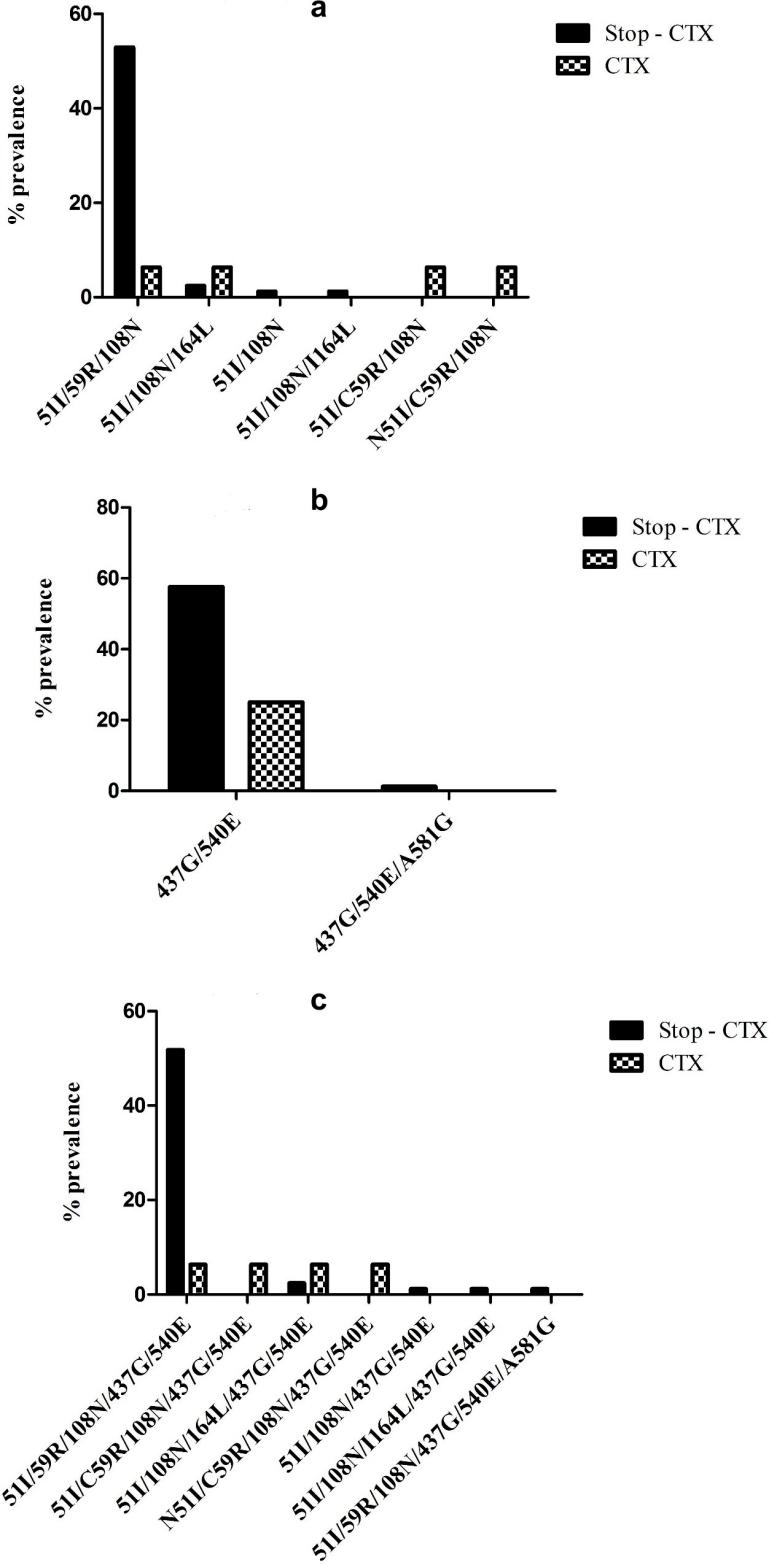
Prevalence of haplotype mutations in *pfdhfr* and *pfdhps* genes in subjects continuing with cotrimoxazole prophylaxis therapy (CTX) and those who stopped CTX therapy (STOP CTX). The prevalence was based on the total number of *P*. *falciparum* positive samples in each arm. The statistical difference in parasite prevalence between the two arms was determined. A) Haplotype mutations (51I, 59R, 108N and 164L) in *pfdhfr* gene; B) haplotype mutations (437G, 540E and 581G) in *pfdhps* gene and; C) haplotype mutations present in both genes. There were statistical significant differences between the STOP CTX and CTX arms in the *pfdhfr* gene haplotype 51I/59R/ 108N (P = 0.0006), in *pfdhps* gene haplotype 437G/540E (P = 0.027) and in both genes haplotype 51I/59R/108N/437G/540E (P = 0.0007).

A total of 2,625 samples were initially screened for presence of malaria parasites by qRT-PCR [17,18]. Of these, 183 samples were positive for *Plasmodium* genus, 131 (71.6%) were *P*. *falciparum*, and 101 (55.2%) were successfully genotyped in *pfdhfr* (at codons 16, 50, 51, 59, 108, and 164) and *pfdhps* (at codons 436, 437, 540, 581, and 613) genes; 30 samples had non-falciparum parasites. The difference in the number of samples that were successfully genotyped by sequencing (n = 101) and those that were *Plasmodium* spp. positive as detected by qRT-PCR (n = 183) is due to the difference in sensitivities of the amplification assays used. For the detection of *Plasmodium* genus used for the initial screening for the presence of the parasite, Ottichilo *et al*. (2016) used previously described qRT-PCR assay (probe based assay) which amplifies total nucleic acids (RNA and DNA) of the 18S rRNA genes, increasing sensitivity several fold [17,18]. For genotypic analysis, nested PCRs that target DNA only [6] were used in the sequencing reactions. Table 1 shows the prevalence of the mutant alleles in *pfdhfr* and *pfdhps* genes. The prevalence was based on the total number of samples that were *P*. *falciparum* positive in each of the arms (total n = 101; STOP-CTX = 85 and CTX = 16). Single nucleotide polymorphisms (SNPs) were designated as wild, mutant or mixed alleles [6,7]. None of the parasite samples contained mutations in *pfdhfr* codons 16 and 50 or in *pfdhps* codons 436 and 613. Three samples, two in STOP-CTX arm and one in the CTX arm had the *pfdhfr* 164L mutation which confers high grade resistance to antifolate [19,20]. The two samples in the STOP-CTX arm were collected in M3 and M12 whereas the one sample in the CTX arm was collected at enrollment. Mutations in the STOP-CTX arm were present at a higher frequency compared to CTX arm in *pfdhfr* codons 51 (65.9% [n = 56/85] vs. 25.0% [n = 4/16]; P = 0.0043), 59 (60% [n = 51/85] vs. 12.5% [n = 2/16]; P = 0.0007) and 108 (65.9% [n = 56/85] vs. 31.3% [n = 5/16]; P = 0.0126). In the *pfdhps* gene, mutations were only present in codons 437 and 540 with frequencies higher in the STOP-CTX arm compared to the CTX arm (68.2% [n = 58/85] vs. 37.5% [n = 6/16] and 70.6% [n = 60/85] vs. 37.5% [n = 6/16] respectively). Fig 1 shows the prevalence of the different mutation haplotypes in *pfdhfr* and/or *pfdhps* genes. The prevalence of the triple mutant haplotype (*pfdhfr* 51I/59R/108N) was 52.9% (n = 45/85) in the STOP-CTX arm versus 6.3% (n = 1/16; P = 0.0006) in the CTX arm, and the *pfdhps* double mutant (437G/540E) was 57.6% (n = 49/85) in the STOP-CTX arm versus 25.0% (n = 4/16; P = 0.027) in the CTX arm. The prevalence of quintuple haplotype (51I/59R/108N/437G/540E) was 51.8% (n = 44/85) in the STOP-CTX arm versus 6.3% (n = 1/16; P = 0.0007) in the CTX arm.

**Table 1:** Prevalence of mutations in the *pfdhfr* and *pfdhps* genes.

		**N51I**		**C59R**		**S108N**		**I164L**	
*DHFR*		STOP-CTX (N = 85)	CTX (N = 16)	STOP-CTX (N = 85)	CTX (N = 16)	STOP-CTX (N = 85)	CTX (N = 16)	STOP-CTX (N = 85)	CTX (N = 16)
	MUTANT	65.9% (56)	25% (4)	60% (51)	12.5% (2)	65.9% (56)	31.3% (5)	2.4% (2)	6.25% (1)
	MIXED	0	6.25% (1)	0	12.5% (2)	0	0	1.2% (1)	0
		**A437G**		**K540E**		**A581G**		**A613S/T**	
*DHPS*		STOP-CTX (N = 85)	CTX (N = 16)	STOP-CTX (N = 85)	CTX (N = 16)	STOP-CTX (N = 85)	CTX (N = 16)	STOP-CTX (N = 85)	CTX (N = 16)
	MUTANT	68.2% (58)	37.5% (6)	70.6% (60)	37.5% (6)	0	0	0	0
	MIXED	0	0	0	0	1.2% (1)	0	0	0

Note: Mutation distribution per codon was calculated as a percentage of the total number of *P*. *falciparum* positive samples in each arm as indicated (N). Mutations were tallied autonomously per codon as an overall prevalence.

### Temporal trends of haplotype mutations in the *pfdhfr* and *pfdhps* genes

**Fig 2:**
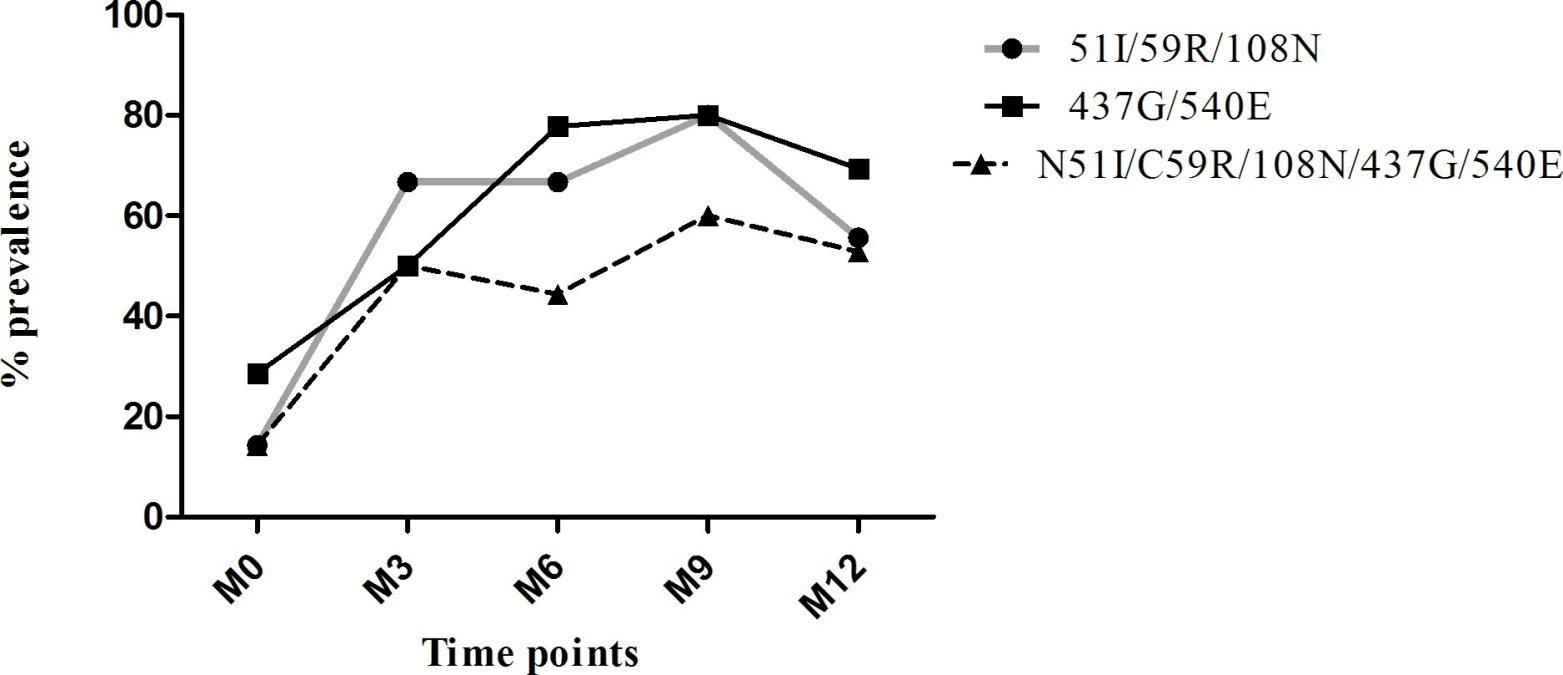
Temporal trends of haplotype mutations in the *pfdhfr* and *pfdhps* genes in the STOP-CTX arm. The prevalence is based on the total number of samples collected at each time point. Temporal trends shown for haplotype mutations in *pfdhfr* gene (51I/59R/108N), *pfdhps* gene (437G/540E) and both genes combined (51I/59R/108N/437G/540E).

To determine change in the prevalence of the mutations over the study period for each study arm, we analyzed samples carrying mutations at each time-point, starting M0 –M12, and the sick visits. However, the sample sizes were small in the CTX arm. In the STOP-CTX arm, the percent prevalence of point mutations in both genes increased over time with marked increase occurring in M9 followed by a slight drop in M12 (Table 2). The difference in prevalence of mutations was less pronounced in the sick visits between the two arms. Fig 2 shows the prevalence of mutations at the different time-points of the triple mutant haplotype (51I/59R/108N) in the *pfdhfr* gene, the double mutant haplotype (437G/540E) in *pfdhps* gene and the quintuple haplotype (51I/59R/108N/437G/540E) in the STOP-CTX arm. All the changes (increases) over time reached statistical significance (P = 0.0069, 95% CI = 19.99–67.45; P = 0.008, 95% CI = 32.90–61.02; and P = 0.0044, 95% CI 18.35–52.09, respectively).

**Table 2:** Temporal change of mutations in the pfdhfr and pfdhps genes.

		M0		M3		M6		M9		M12		SICK	
		STOP (N = 7)	CTX (N = 4)	STOP (N = 6)	CTX (N = 1)	STOP (N = 9)	CTX (N = 1)	STOP (N = 15)	CTX (N = 3)	STOP (N = 36)	CTX (N = 3	STOP (N = 12)	CTX (N = 4)
DHFR	N51I	28.6% (2)	25.0% (1)	66.7% (4)	0.0	66.7% (6)	0.0	93.3% (14)	33.3% (1)	63.9% (23)	0.0	58.3% (7)	50.0% (2)
	C59R	14.3% (1)	0.0	66.7% (4)	0.0	66.7% (6)	0.0	80.0% (12)	33.3% (1)	58.3% (21)	0.0	58.3% (7)	25.0% (1)
	S108N	0.0	25% (1)	66.7% (4)	0.0	66.7% (6)	0.0	93.3% (14)	0.0	63.9% (23)	0.0	58.3% (7)	0.0
	I164L	0.0	25% (1)	0.0	0.0	0.0	0.0	6.7% (1)	0.0	2.8% (1)	0.0	0.0	0.0
DHPS	A437G	28.6% (2)	50.0% (2)	50.0% (3)	0.0	77.8% (7)	0.0	80.0% (12)	33.3% (1)	66.7% (24)	0.0	83.3% (10)	75.0% (3)
	K540E	28.6% (2)	50.0% (2)	50.0% (3)	0.0	88.9% (8)	0.0	80.0% (12)	33.3% (1)	69.4% (25)	0.0	83.3% (10)	75.0% (3)
	A581G	0.0	0.0	0.0	0.0	77.8% (7)	0.0	0.0	0.0	0.0	0.0	0.0	0.0
DHFR HAPLOTYPES	51I/59R/108N	14.3% (1)	0.0	66.7% (4)	0.0	66.7% (6)	0.0	80.0% (12)	33.3% (1)	55.6% (20)	0.0	58.3% (7)	25.0% (1)
DHPS HAPLOTYPES	437G/540E	28.6% (2)	50.0% (2)	50.0% (3)	0.0	77.8% (7)	0.0	80.0% (12)	33.3% (1)	69.4% (25)	0.0	83.3% (10)	75.0% (3)
DHFR/DHPS HAPLOTYPES	51I/59R/108N/437G/540E	14.3% (1)	0.0	50.0% (3)	0.0	44.4% (4)	0.0	60.0% (9)	0.0	52.8% (19)	0.0	58.3% (7)	25.0% (1)

Data shows the prevalence of mutations at each time point, calculated as a percentage of the total number of *P*. *falciparum* positive samples at each time-point for each arm as indicated (N).

## Discussion

The clinical benefits of using cotrimoxazole prophylaxis in controlling opportunistic infections including falciparum malaria in HIV-infected individuals are clear [21]. The widespread use of cotrimoxazole prophylaxis for individuals with HIV infection in malaria endemic countries has been a concern because of the risk of developing cross resistance to other antifolate drugs [22–24]. However, concern for cross resistance was based on in vitro data [3,25,26], and has not been substantiated by field clinical data (reviewed by [27]). Despite the high prevalence of SP-resistant mutations, cotrimoxazole continues to provide important benefits in reducing morbidity and mortality particularly in the setting of HIV infection [28–30], and does not lead to increased resistance [22,31–33]. Current field data clearly supports the continued use of cotrimoxazole as a prophylactic drug in HIV-infected populations [21,27]. Additional studies to investigate the use of cotrimoxazole as an alternative to SP in IPTp/i, malaria treatment and prophylaxis, and as a combined anti-malarial therapy with artemisinin are warranted.

In this study, we demonstrated cotrimoxazole prophylaxis did not results in increased risk of developing resistance, corroborating previous studies [22,28–32,34]. Interestingly, we found the prevalence of SP-resistant alleles increased steadily over the study period in the STOP-CTX arm for the first 9 months. Further, although the sample size was small, the prevalence of SP-resistant alleles in the CTX arm did not change over the study period. Taken together, cotrimoxazole prophylaxis lowered the overall incidence of SP-resistant parasites, consistent with previous studies [22,33]. Key SP-resistant mutations have become fixed in parasite populations despite discontinuation of SP as the first-line treatment for more than a decade [6,7,9], indicating these mutations might be providing benefit to the parasite population without fitness cost. It is possible that cotrimoxazole selects against parasites carrying SP-resistant alleles in the population, and removal of cotrimoxazole pressure allows the SP-resistant parasite population, which seems to be more fit than SP-susceptible population to dominate. As studies are underway to investigate expanded role of cotrimoxazole in developing countries [21], the use of cotrimoxazole in the prevention of malaria HIV-infected and HIV uninfected populations, especially as a tool for malaria elimination and as travelers’ prophylactic drug, needs to be further investigated.

Mutation in *pfdhfr* 164L codon is associated with high grade resistance to SP [35–38]. While some studies have shown evidence that cotrimoxazole prophylaxis might be associated with presence of *pfdhfr* 164L codon [31,39], other studies do not support this observation [40]. In Kenya, only low prevalence of *pfdhfr* 164L has been reported [6,7,36,41]. In our study, three parasite isolates had *pfdhfr* 164L, one in CTX arm collected at M0 and two in STOP-CTX collected in M9 and M12, indicating the presence of this mutation is unlikely due to cotrimoxazole drug pressure. Although cotrimoxazole has been speculated to contribute to antifolate selective pressure [40], additional studies are required to support this observation.

This study had several limitations. First, the study was unblinded clinical trial without a placebo or concurrent control group of HIV-uninfected individuals [16]. Second, the number of infections in the CTX arm was small, limiting statistical analysis or might have resulted in bias. This made it especially difficult in interpreting the data when evaluating prevalence at each time-point. Also, the use of highly sensitive qRT-PCR assay for the initial screening followed by multiple sequencing reactions, which uses multiple PCR steps that are not as sensitive was a limitation.

In conclusion, this study demonstrated cotrimoxazole does not select for SP-resistance *P*. *falciparum* parasites but instead, lowers the overall incidence of SP-resistant parasites. Since cotrimoxazole is available in malaria-HIV co-endemic regions with infrastructure in place and is effective against SP-resistant parasites, additional studies are required to validate these findings and to further explore the possibility of expanding the use of this drug for IPTp/i, prophylaxis for non-HIV population and travelers.

## Supporting information

S1 Fig Trial profile. Consort figure from Polyak et al., (Ref: 16) showing study retention. A total of 490 participants (98%) were retained to the end of scheduled follow-up. Participants randomized to the CTX continuation arm self-reported that they took CTX every day in the past week at 90.5% of follow-up visits.
(PDF)
