# Supplementary material for: The prevalence and antifolate drug resistance profiles of Plasmodium falciparum in study participants randomized to discontinue or continue cotrimoxazole prophylaxis
Source: PLoS Negl Trop Dis. 2019 Mar 21;13(3):e0007223. doi: 10.1371/journal.pntd.0007223 (PMC6445470; doi:10.1371/journal.pntd.0007223)
Supplement: S1 Fig — Consort figure from Polyak et al., (Ref: 16) showing study retention. A total of 490 participants (98%) were retained to the end of scheduled follow-up. Participants randomized to the CTX continuation arm self-reported that they took CTX every day in the past week at 90.5% of follow-up visits. (PDF) [file pntd.0007223.s001.pdf]

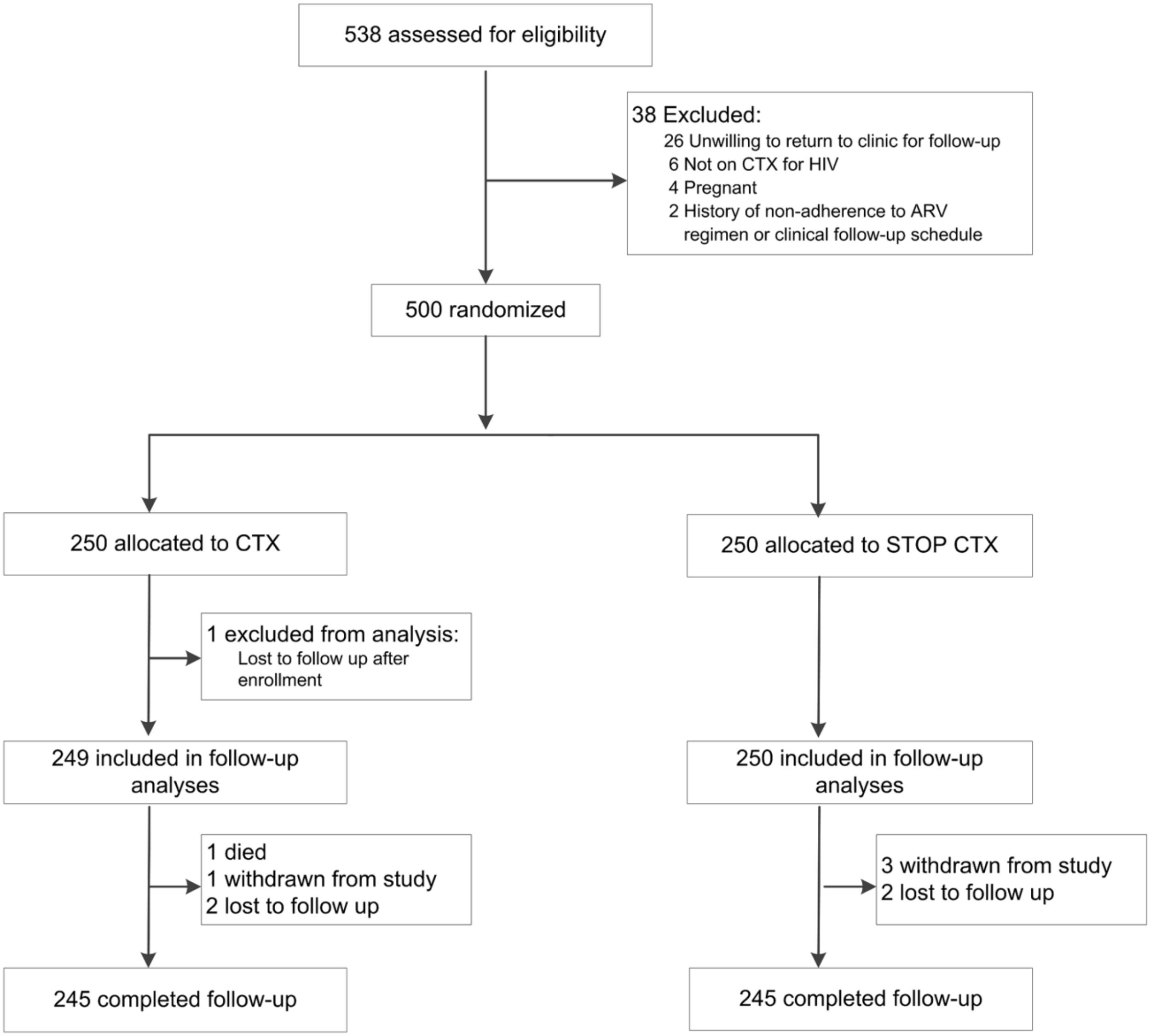

Figure S1. Trial profile.

Consort figure from Polyak et al., (Ref: 16) showing study retention. A total of 490 participants (98%) were retained to the end of scheduled follow-up. Participants randomized to the CTX continuation arm self-reported that they took CTX every day in the past week at 90.5% of follow-up visits.
